# Effect of stabilizer on dynamic thermal transport property of ZnO nanofluid

**DOI:** 10.1186/1556-276X-8-125

**Published:** 2013-03-14

**Authors:** Rajesh Kumar Neogy, Arup Kumar Raychaudhuri

**Affiliations:** 1 Unit for Nanoscience, Department of Condensed Matter Physics and Material Sciences, S. N. Bose National Centre for Basic Sciences, Sector-III, Block-JD, Salt Lake, Kolkata 700098, India

**Keywords:** Heat transport, Nanofluid, ZnO nanoparticles, Stabilizer

## Abstract

In this paper, we investigate the effect of adding a stabilizer on the dynamic thermal properties of ZnO nanofluid (containing 5 to 10 nm diameter of ZnO nanocrystals) measured using a 3*ω* method. Addition of the stabilizer leads to the stabilization of the nanofluid and also substantial reduction of the enhancement of thermal transport compared to that seen in the bare ZnO nanofluid. This also alters the frequency dependence of the thermal transport and the characteristic time scale associated with it. It is suggested that the addition of the stabilizer inhibits the thermodiffusion-assisted local aggregation thus leading to substantial reduction of the enhancement of thermal transport properties of the bare nanofluid as proposed in some recent models, and this also alters the characteristic time scales by altering the scale of aggregation.

## Background

Nanofluids are dispersions of nanoparticles (typically sizes approximately 5 to 20 nm) in liquid medium. In recent years, they have attracted considerable attention due to enhanced heat transport properties as seen through enhanced thermal conductance [1,2]. In general, heat transport due to conducting metallic or solid inclusions in nonconducting fluids leads to an enhancement. However, in the nanofluids, which have solid inclusions of sizes in the range of few nanometers or few tens of nanometers, the enhancement in thermal conductivity was found to be much larger than that expected from Maxwell’s effective medium theories [3,4].

A number of mechanisms have been proposed that could be responsible for the enhancement of the thermal conductivity. They include the (a) Brownian motion of the nanoparticles [5,6], (b) molecular-level layering of the liquid at the liquid-particle interface [7], (c) ballistic heat transport in nanoparticles [8], and (d) local clustering of nanoparticles [9,10]. The suggested mechanisms do provide some level of explanation of the enhancement. However, there is no accepted theory/mechanism that can explain all the observations adequately.

Recently reported experimental studies suggest that the formation of local nanoparticle aggregate can play a significant role in the thermal transport in nanofluids [9,10]. In the context of nanofluids containing Fe nanoparticles, it was demonstrated [11] that Fe nanoparticles in the nanofluids can locally assemble into aggregate of micron-size clusters. It was found in CuO nanofluids that large thermal conductivity enhancements are often accompanied by sharp viscosity that increases at low nanoparticle volume fractions, which has been inferred as an indicative of local aggregation effects [12]. The aggregation can be controlled by surface charge, and the critical importance of particle surface charge in nanofluid thermal conductivity has been demonstrated [13].

In this paper, we carry out an investigation on the effect of local aggregation on the thermal transport in nanofluids. This was done in nanofluids containing ZnO nanoparticles with and without stabilizer. The stabilizer can affect local aggregation which in turn can substantially change the enhancement of the thermal conduction in nanofluids. Importantly, we also show that this affects the characteristic frequency scales associated with the dynamical heat transport in such nanofluids. The characteristic frequency, as derived directly from the dynamic thermal property measurement, is a measure of the scale of local aggregation. The reduction of the scale of local aggregation can reduce the magnitude of the thermal transport enhancement, providing a direct link between the two.

The choice of ZnO nanofluid for the investigation originates from the fact that unlike many metallic nanofluids, ZnO nanofluids can be a stable suspension over hours even without added stabilizers. This stability arises due to surface charges on as-prepared ZnO nanoparticles [14]. The stability over hours is long enough that it enables us to carry out the thermal measurements. The addition of polyvinylpyrrolidone (PVP) as a stabilizer enhances the stability even further to weeks and even months. Thus, the system chosen is a very suitable system where the measurements can be carried out in nanofluids with and without stabilizers and thus track the changes in thermal parameters in the addition of the stabilizer.

In our earlier work on ZnO nanofluids [15], which is carried out using a dynamic 3*ω* technique, it has shown that the parameter effusivity (*C*_*p*_*κ*, *C*_*p*_ = heat capacity, *κ* = thermal conductivity) has a prominent frequency dependence. The measured effusivity shows appreciable enhancement at low frequency, but above a characteristic frequency, the enhancement is significantly reduced and it approaches the parameters of the base liquid. In this paper, we investigate what happens to the enhancement of *C*_*p*_*κ* as well as its frequency dependence when a stabilizer is added to the system. We find that the presence of stabilizer, which reduces the local aggregation, actually leads to a significant decrease of the *C*_*p*_*κ*. We also find that the frequency dependence of *C*_*p*_*κ* in bare ZnO nanofluid gets quantitatively modified when the stabilizer is attached. In addition, we carry out an analysis of the frequency dependence of the temperature oscillation to separate out the contributions of *C*_*p*_ and *κ* components and find that the enhancement in *C*_*p*_*κ* is primarily due to the enhancement of thermal conductivity *κ*.

## Methods

### Nanofluid synthesis

**Figure 1:**
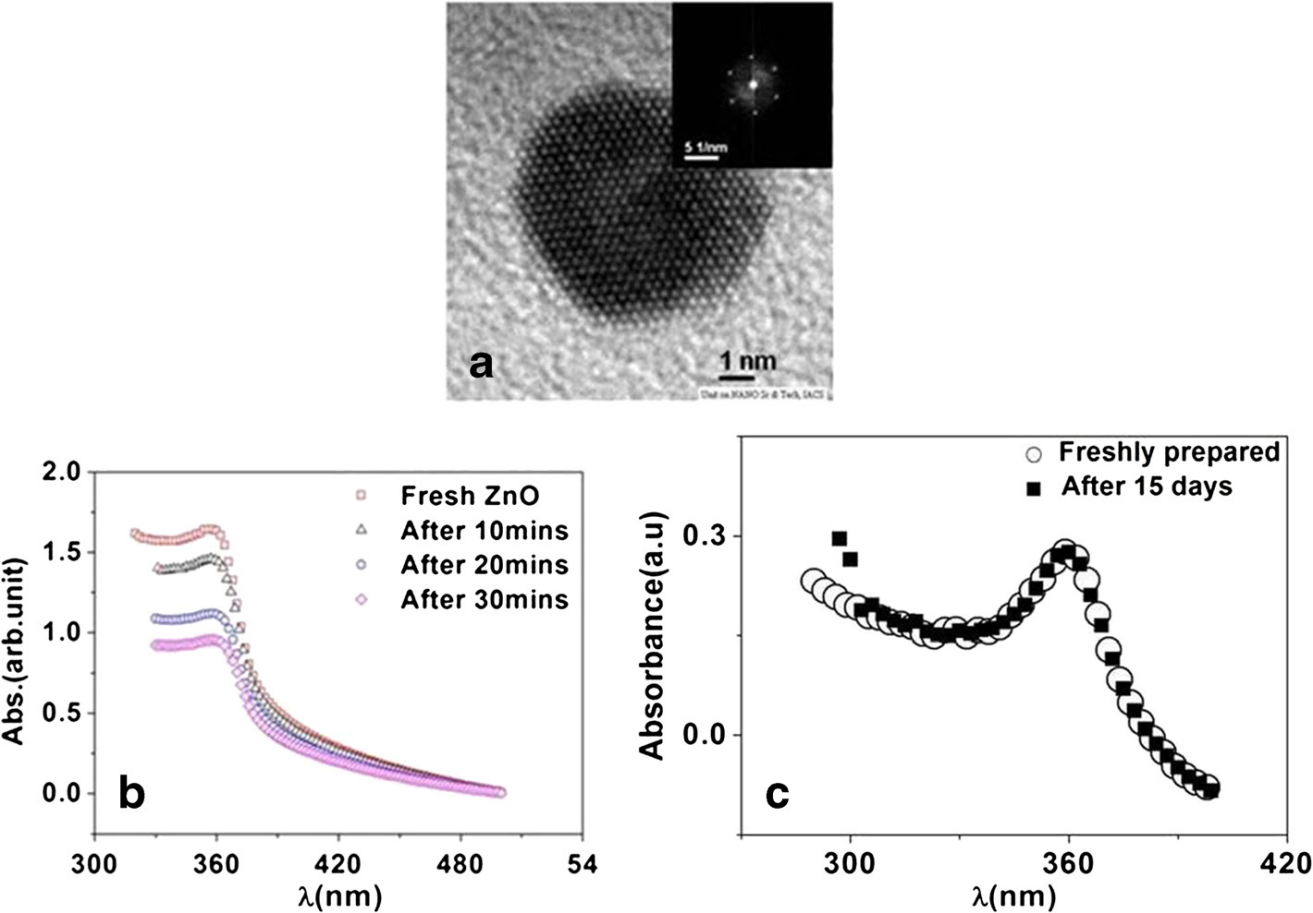
**TEM image of ZnO nanocrystal used. **(**a**) Time dependence of UV–vis spectra for ZnO nanofluids, (**b**) without and (**c**) with PVP stabilizer.

Stable ZnO nanofluid, which is a dispersion of ZnO nanocrystals in ethanol, is prepared by wet chemical method [16]. The nanocrystals of ZnO were synthesized at low temperature (<90°C) in an alkaline medium using Zn acetate. The nanocrystals of ZnO are crystalline with an average size of approximately 10 nm as seen using the transmission electron microscope (TEM). The typical TEM image of a nanoparticle is shown in Figure 1a. Two nanofluids were prepared. One is a pure dispersion of the ZnO nanocrystals in ethanol, and the other is made by adding PVP as a stabilizer. PVP binds to the polar surface of ZnO. The ZnO nanofluid, even without PVP, can be stabilized in scales of hours. The addition of PVP leads to substantial enhancement of the stability of the nanofluid. PVP has been used in the past to make stable metal colloids of Pd [17], Au, and Ag [18]. We have monitored the stability of the nanofluids by measuring the UV–vis spectra of the fluids as a function of time. Both the nanofluids show characteristic absorption around *λ* ≈ 360 nm, which is the absorption edge for ZnO. For the ZnO nanofluid without PVP, the absorption initially decreases with time as shown in Figure 1b. The decrease is rapid initially and then slows down considerably after 30 min, when the absorption decreases by about 3% in 1 h. This stability is long enough to carry out the thermal measurements over a period of 2 h. The addition of the PVP leads to a very stable nanofluid that is stable over few weeks as can be seen in Figure 1c where there is no perceptible change in the UV-visible absorption even after 2 weeks.

### Thermal measurements using 3*ω* technique

The thermal measurements were done using a 3*ω* technique [19-21], where we use a platinum film both as a thermometer and a heater. The method, as applied to nanofluids, is explained elsewhere [15]. Here, we provide a small gist for quick reference.

In this method, the Pt film (width of 300 μm, thickness of 50 nm, and length of 5 mm grown on a glass substrate by magnetron sputtering) carrying a current at frequency *f* is immersed in the liquid in which measurements have to be made [19]. The periodic heating of the film, due to the sinusoidal current, makes the temperature oscillate around the average with an amplitude *δT*_2*ω*_ at a frequency 2*ω* (*ω* = 2*πf*). This leads to resistance oscillations of amplitude *δT*_2*ω*_ at frequency 2*ω* around the mean, where *δR*_2*ω*_ = *αR*_0_*δT*_2*ω*,_*α* is the temperature coefficient of resistance (TCR) of the heater, and *R*_0_ is the average resistance of the heater. The resistance oscillation *δR*_2*ω*_ at frequency 2*ω* mixes with the current at frequency *ω* to produce a potential drop (${\tilde{V}}_{3\omega }$) with a component at 3*ω* (sum band). The experiment measures the complex voltage ${\tilde{V}}_{3\omega }$ with its phase and amplitude, using a phase-sensitive detection technique. The thermal properties of the heater-on-substrate (S) and surrounding liquid (L) are given by two parameters *Z* and the phase *φ*. These parameters are obtained experimentally from the observed 3*ω* signal ${\tilde{V}}_{3\omega }$, the area of the heater (*A*), the power dissipated (*P*), and the measured TCR (*α*) of the Pt film using the equation [19]

(1) $$Ze^{\mathrm{i\phi}}=\frac{e^{-\mathrm{i\phi}}}{\sqrt{\xi_{S}+\sqrt{\xi_{L}}}}=\frac{2A}{\mathrm{\alpha P}}\sqrt{2\omega }{\tilde{V}}_{3\omega },$$

where the thermal parameter is the effusivity given as *ξ* ≡ *C*_*p*_*κ*. L and S refer to the liquid and the substrate, respectively.

The Pt film has a resistance of ≈ 100 Ω and a measured temperature coefficient of resistivity *α* ≈ 3.5 × 10^−3^/K. The relative size of the heater width and the thermodiffusion length $\lambda_{\mathrm{th}}=\sqrt{D/2\omega }$ (*D* = thermal diffusivity) determines the low-frequency range of the experiment. In our case for the base liquid ethanol (*D* ≈ 9 × 10^−8^ m^2^/s), the working frequency is $1\;\mathrm{Hz}<\frac{\omega }{2\pi }<1\;\mathrm{kHz}$ for the width of the heater used (approximately 300 μm). At high-frequency range, the limit arises due to the low value of the signal. The experiment was carried out in a temperature-controlled bath stabilized to better than ±0.01 K which houses a cylindrical copper shell as the sample container. The typical data-taking time for a given frequency scan over the full range is 30 min. After each scan, the suspension is shaken in an ultrasonic shaker before the next run begins.

Using relation $\left|Z_{s}\right|=\frac{1}{\sqrt{\xi_{S}}}$ and $\left|Z_{\mathrm{NF}}\right|=\frac{1}{\sqrt{\xi_{S}}+\sqrt{\xi_{\mathrm{NF}}}}$, we obtain the *ξ*_NF_ for the nanofluid given as [19]

(2) $${\left(C_{p}\kappa \right)}_{\mathrm{NF}}=\xi_{\mathrm{NF}}={\left[\frac{Z_{S}-Z_{\mathrm{NF}}}{Z_{\mathrm{NF}}}\right]}^{2}{\left(\xi \right)}_{S}.$$

In addition to the effusivity *ξ*_NF_, we also find the thermal conductivity *κ* using the frequency dependence of the temperature oscillation *δT*_2*ω*_. The *δT*_2*ω*_ for a line heater has a total width of 2*b* dissipating power *P*_*L*_/unit length and immersed in a liquid [20]:

(3) $$\delta T_{2\omega }=\frac{P_{L}}{\pi \kappa }\overset{\infty }{\underset{0}{\int }}\frac{\sin^{2}\left(\mathrm{Kb}\right)}{{\left(\mathrm{Kb}\right)}^{2}}\frac{1+\gamma_{s}}{\gamma_{l}+\gamma_{s}+\gamma_{s}\gamma_{l}R_{\mathrm{interface}}}\mathrm{dK},$$

where *K* is the integration variable, q=2ωρCpκi, $\gamma_{j}=\kappa_{j}\sqrt{K^{2}+q_{j}^{2}}\;j=s,l$ refer to the solid (substrate-carrying heater) and the liquid, respectively. The value of the interfacial resistance is expressed as *R*_interface_ ≈ 6.1 × 10^−7^ m^2^ K/W [20]. From Equation 4, it can be shown that the frequency dependence of *δT*_2*ω*_ has a logarithmic dependence on *f* whose slope is given as [21]

(4) $$\kappa =\left(\frac{-P_{l}}{2\pi }\right){\left(\frac{\partial T_{2\omega }}{\partial \ln\omega }\right)}^{-1}.$$

We also determine the specific heat *C*_*p*_ of the base liquid and the nanofluids using a differential scanning calorimeter, operating in modulation mode (with frequency <10 mHz).

## Results and discussions

### Change in thermal effusivity in the addition of stabilizer

**Figure 2:**
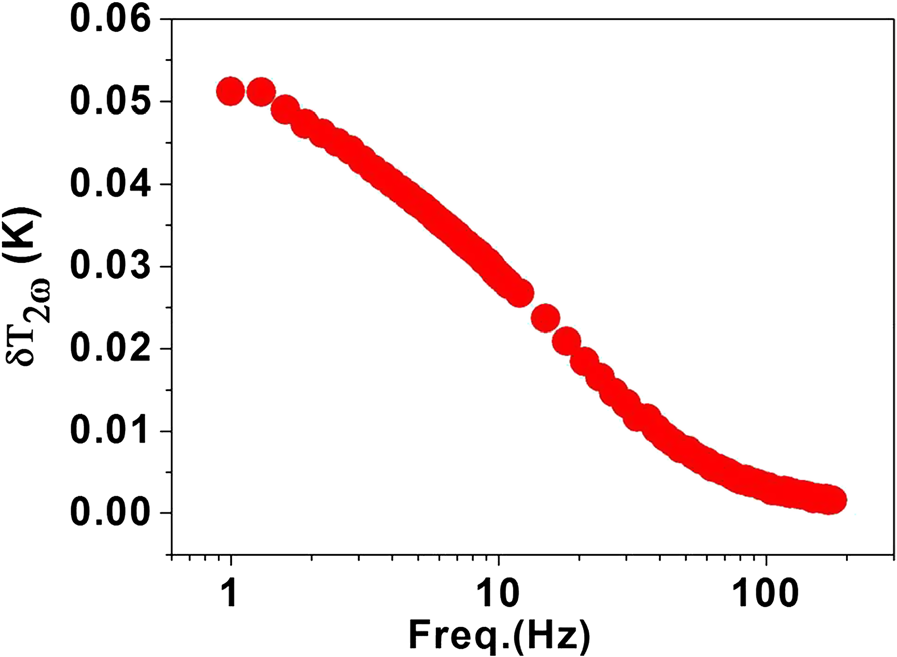
**Typical temperature oscillation *δT***_**2*ω***_**as a function of frequency measured in PVP-stabilized ZnO nanofluid.**

The representative data on the detected temperature oscillation *δT*_2*ω*_ as a function of frequency is shown in Figure 2. It shows the typical *δT*_2*ω*_ data for ZnO-PVP nanofluids. From this data, we do the analysis of thermal conductivity of respective nanofluids.

**Figure 3:**
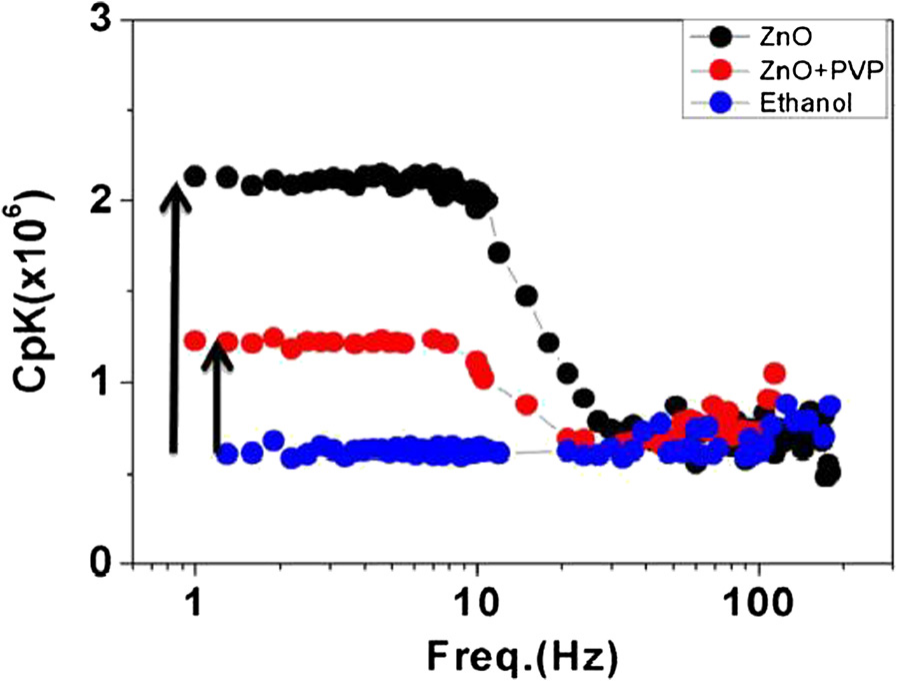
Frequency dependence of effusivity of base liquid ethanol, bare ZnO nanofluid, and PVP-stabilized ZnO nanofluid.

In Figure 3, we show the effusivity *ξ*_NF_ = *C*_*p*_*κ* of the base fluid ethanol along with two nanofluids: the bare ZnO nanofluid as well as the ZnO nanofluid with stabilizer PVP. The data for the base liquid ethanol are also shown. The parameters are obtained from Equations 2 and 4 using the measured data. Both the nanofluids have the same volume fraction of 1.5% and have similar average particle size.

The enhancement of *ξ*_NF_ in the nanofluids, at low frequency, compared to that in ethanol is clearly seen. Importantly, it is observed that the enhancement in the bare nanofluid (without stabilizer) is much larger compared with that in the nanofluid with the PVP stabilizer. The results are summarized in Table 1, where we show the enhancement of the effusivity *ξ* = *C*_*p*_*κ* as a ratio taken with respect to (wrt) the base fluid as determined from the analysis of the ${\tilde{V}}_{3\omega }$ signal. The low-frequency-limiting values for *ξ* were used for the parameters in Table 1.

**Table 1:** Comparison of thermal parameters for nanofluids as measured by two methods

**Quantity/method**	**Bare ZnO nanofluid**	**ZnO nanofluid with PVP**
Relative enhancement of effusivity *ξ* = *C*_*p*_*κ* wrt ethanol/from 3*ω* method using ${\tilde{V}}_{3\omega }$	4.0	2.7
Relative enhancement of thermal conductivity *κ* wrt ethanol/from temperature oscillation *δT*_2*ω*_	4.2	2.4

The value of the measured specific heat *C*_*p*_ of the base fluid as well as the nanofluids are comparable (*C*_*p*_ ≈ 2.5 J/g K). It is thus clear that the enhancement of the effusivity in both the nanofluids is arising primarily due to the enhancement of the thermal conductivity *κ*.

To make an independent check on the enhancement of the thermal conductivity, we used the measured frequency dependence of the thermal oscillation *δT*_2*ω*_. Equation 4 gives a limiting low-temperature slope for *δT*_2*ω*_ wrt the frequency (log *f*) that is proportional to *κ*^−1^. Using this information, we obtain the relative enhancement of the thermal conductivity wrt the base fluid ethanol. The data for both the nanofluids are shown in Table 1. It can be seen that this also gives nearly the same value for enhancement (within 15% to 20%), which confirms that there is indeed an enhancement in *κ* in the nanofluids. It is gratifying that the analysis from both the parameters *δT*_2*ω*_ and ${\tilde{V}}_{3\omega }$ gives similar results.

It can be seen from Table 1 that the enhancement *κ* for the bare ZnO nanofluid is significantly larger than that seen in the PVP-stabilized ZnO nanofluid. This gives us the first important result that there is indeed a significant reduction in the effusivity and thermal conductivity on stabilizing the ZnO nanofluid with stabilizer that inhibits the local aggregation significantly, which in turn leads to its long-term stability. This observation establishes a direct connection between the enhancement of *κ* and the local aggregate formation.

### The frequency dependence of the enhancement and its analysis

The enhancement of the effusivity in nanofluids has a frequency dependence as shown in Figure 3, where the enhancement decreased at higher frequency, and for *f* > 30 Hz, the values of *C*_*p*_*κ* for both the nanofluids approach that of the base fluid ethanol. This frequency dependence of the effusivity for bare ZnO nanofluid (without PVP) has been reported elsewhere [15]. It was proposed that the frequency dependence can arise from dynamic local aggregation. In this paper, we explore the proposed hypothesis whether the frequency dependence indeed has a connection to the local aggregation. At low frequency (*f* ≤ 10 Hz), the enhancement is large, and it reaches a frequency-independent value.

The decrease in the effusivity at higher frequency in both the nanofluids can be fitted by the low-pass filter relation:

(5) $$\frac{{\left(C_{p}\kappa \right)}_{\mathrm{NF}}\left(f\right)}{{\left(C_{p}\kappa \right)}_{\mathrm{NF}}\left(f=0\right)}=\frac{1}{\sqrt{1+{\left(\frac{f}{f_{c}}\right)}^{2n}}}.$$

The corner frequency *f*_*c*_ and the order of the filter *n* can be obtained from the fit to the data. For the ZnO nanofluid without PVP, the data can be fitted by the first-order filter function (*n* = 1). For fluid with PVP, we got a different higher order value, which is *n = 5*.

**Figure 4:**
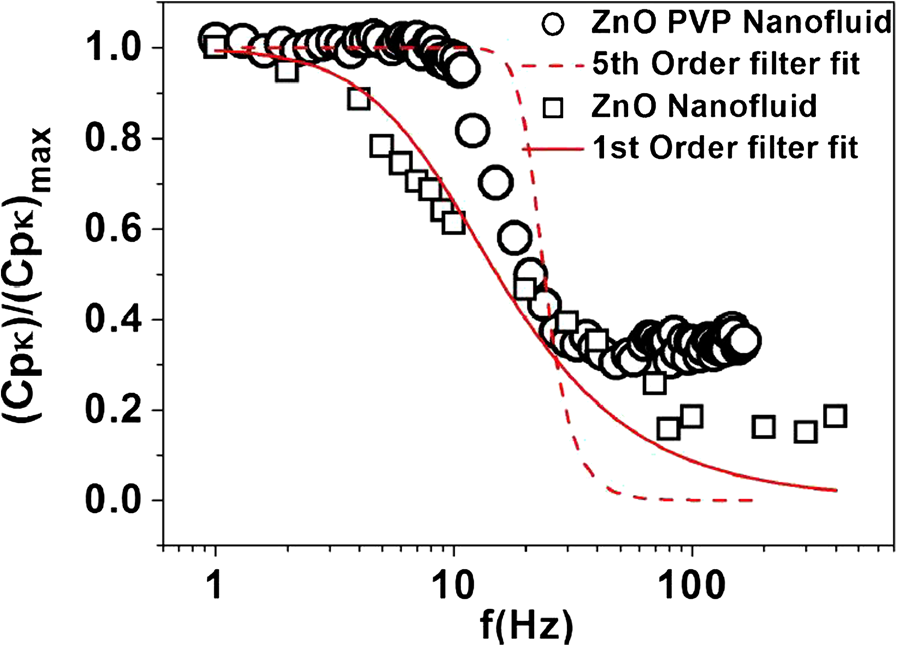
Low-pass filter response fit for ZnO nanofluids and ZnO-PVP nanofluid.

In Figure 4, we show the fit of the data to Equation 5. The data for both the nanofluids are shown.

The data are summarized in Table 2. From the table, it can be seen that there is a significant reduction in the corner frequency *f*_*c*_ in the addition of the stabilizer.

**Table 2:** Corner frequency, relaxation time, and estimated length scale of local agglomeration obtained from the data

**Nanofluid system**	***f***_***c ***_**(Hz)**	***τ *(ms)**	***L***_***A ***_**(μm)**
ZnO	23 ± 1.5	4 ± 3	18 ± 2
ZnO + PVP	43 ± 2.3	2 ± 1	13 ± 2

The thermally driven local aggregation, which would enhance the local thermal transport and hence the value of the thermal conductivity, would lead to solid-like aggregated region in the nanofluids. It is proposed that the response of the type shown in Equation 5 is a manifestation of this local aggregation. The local aggregates respond to an oscillating temperature field *δT*_2*ω*_ with a characteristic thermal relaxation time *τ*_*c*_. This will be related to the characteristic length scales of the local aggregate *L*_*A*_ through the thermal diffusivity *D* by the relation *τ*_*c*_ ≈ *D*^−1^*L*_*A*_^2^. The relaxation time will determine the corner frequency *f*_*c*_ ≈ (4*πτ*_*c*_)^−1^ (the extra factor of 2 arises because the temperature oscillation is at frequency 2*f*). For frequencies larger than 2*f*_*c*_, the temperature oscillation is too fast for the aggregate to respond leading to a decrease in the enhancement of heat transport.

In Table 2, we show the characteristic time *τ*_*c*_ as well as the aggregation length *L*_*A*_ as derived from the data. We find that the addition of the stabilizer leads to the reduction of the aggregation length *L*_*A*_ by 25% to 30%. The corresponding reduction in effusivity or the thermal conductivity is around 40%. This agrees well with the hypothesis that the local aggregation can control the enhancement of the thermal transport as well as the frequency response.

## Conclusions

We have investigated the dynamical thermal property (effusivity and thermal conductivity) of ZnO nanofluids containing ZnO nanocrystals with an average diameter of 10 nm with and without PVP stabilizer. This was done to investigate the role of the stabilizer in the enhancement of thermal transport properties of nanofluids. It had been suggested that thermodiffusion-assisted ‘solid-like’ local aggregation of the nanoparticles in the nanofluids can be the origin of enhancement of thermal conductivity in nanofluids. The investigations carried out on bare ZnO nanofluids as well as PVP-stabilized nanofluids show that addition of a stabilizer, which inhibits diffusion-assisted local aggregation due to attached moiety, leads to reduction in the enhancement of thermal parameters that are observed in bare ZnO nanofluids. It has also been shown, from characteristic time scales of the dynamic thermal measurements, that the scale of aggregation gets reduced in the addition of stabilizers. The experimental results provide evidence that the origin of enhancement of thermal conductivity in nanofluids can arise from local aggregation that occurs by thermodiffusion.

## Competing interests

The authors declare that they have no competing interests.

## Authors’ contributions

RKN and AKR jointly did the planning of the experiment, analysis of the data, and writing the manuscript. RKN did the synthesis, characterization, and the measurements. Both authors read and approved the final manuscript.
